# Alternated selection mechanisms maintain adaptive diversity in different demographic scenarios of a large carnivore

**DOI:** 10.1186/s12862-019-1420-5

**Published:** 2019-04-11

**Authors:** Rita G. Rocha, Vanessa Magalhães, José V. López-Bao, Wessel van der Loo, Luis Llaneza, Francisco Alvares, Pedro J. Esteves, Raquel Godinho

**Affiliations:** 1 CIBIO/InBio - Centro de Investigação em Biodiversidade e Recursos Genéticos, Universidade do Porto, Campus de Vairão, 4485-661 Vairão, Portugal; 2 Research Unit of Biodiversity (UO/CSIC/PA), University of Oviedo, 33600 Mieres, Spain; 3 A.RE.NA, S.L. Asesores en Recursos Naturales S.L., 27003 Lugo, Spain; 4 Departamento de Biologia, Faculdade de Ciências, Universidade do Porto, 4169-007 Porto, Portugal

**Keywords:** *Canis lupus*, Demography, Major histocompatibility complex, Microsatellites, Selection mechanisms

## Abstract

**Background:**

Different population trajectories are expected to impact the signature of neutral and adaptive processes at multiple levels, challenging the assessment of the relative roles of different microevolutionary forces. Here, we integrate adaptive and neutral variability patterns to disentangle how adaptive diversity is driven under different demographic scenarios within the Iberian wolf (*Canis lupus*) range. We studied the persistent, the expanding and a small, isolated group within the Iberian wolf population, using 3 MHC class II genes (DRB1, DQA1, and DQB1), which diversity was compared with 39 microsatellite loci.

**Results:**

Both the persistent and the expanding groups show evidence of balancing selection, revealed by a significant departure from neutrality at MHC loci, significant higher observed and expected heterozygosity and lower differentiation at MHC than at neutral loci, and signs of positive selection. However, despite exhibiting a significantly higher genetic diversity than the isolated group, the persistent group did not show significant excess of MHC heterozygotes. The expanding group, while showing a similar level of genetic diversity than the persistent group, displays by contrast a significant excess of MHC heterozygotes, which is compatible with the heterozygote advantage mechanism. Results are not clear regarding the role of drift and selection in the isolated group due to the small size of this population. Although diversity indices of MHC loci correspond to neutral expectations in the isolated group, accelerated MHC divergence, revealed by a higher differentiation at MHC than neutral loci, may indicate diversifying selection.

**Conclusion:**

Different selective pressures were observed in the three different demographic scenarios, which are possibly driven by different selection mechanisms to maintain adaptive diversity.

**Electronic supplementary material:**

The online version of this article (10.1186/s12862-019-1420-5) contains supplementary material, which is available to authorized users.

## Background

Genetic drift and natural selection are often seen as two counteracting evolutionary forces across populations [1]. In large and stable populations, where the random effects of genetic drift are limited, balancing selection maintains high levels of adaptive variation affecting population fitness and evolutionary potential [2, 3]. By contrast, in small and isolated populations, or within the context of demographic events such as bottlenecks, genetic drift is predominant, masking possible effects of natural selection [4, 5]. Neutral molecular markers allow assessing interactions between demographic history and genetic diversity [6], but do not provide direct information on selective processes [7, 8]. Thus, evidence of selection and adaptive potential is best approached by contrasting neutral and functional molecular markers (e.g., [9, 10]).

In vertebrates, the major histocompatibility complex (MHC) is the prime candidate for pathogen resistance genes and contains some of the most polymorphic functional loci [11, 12]. The exceptional level of MHC polymorphism is believed to be driven by the antagonistic coevolution with pathogens and occurs through pathogen-mediated balancing selection [11, 12]. Beyond its clear significance in modulating pathogen resistance [2, 13, 14], MHC genes have been shown to influence other biological traits such as maternal-fetal interactions, kin recognition, life-time reproductive success and mate choice [15–18]. MHC is intimately linked with factors likely to affect individual fitness, population viability and evolutionary potential in changing environments. Thus, patterns of MHC diversity have been repeatedly used in a conservation context in populations of particular interest [19–22].

**Fig. 1:**
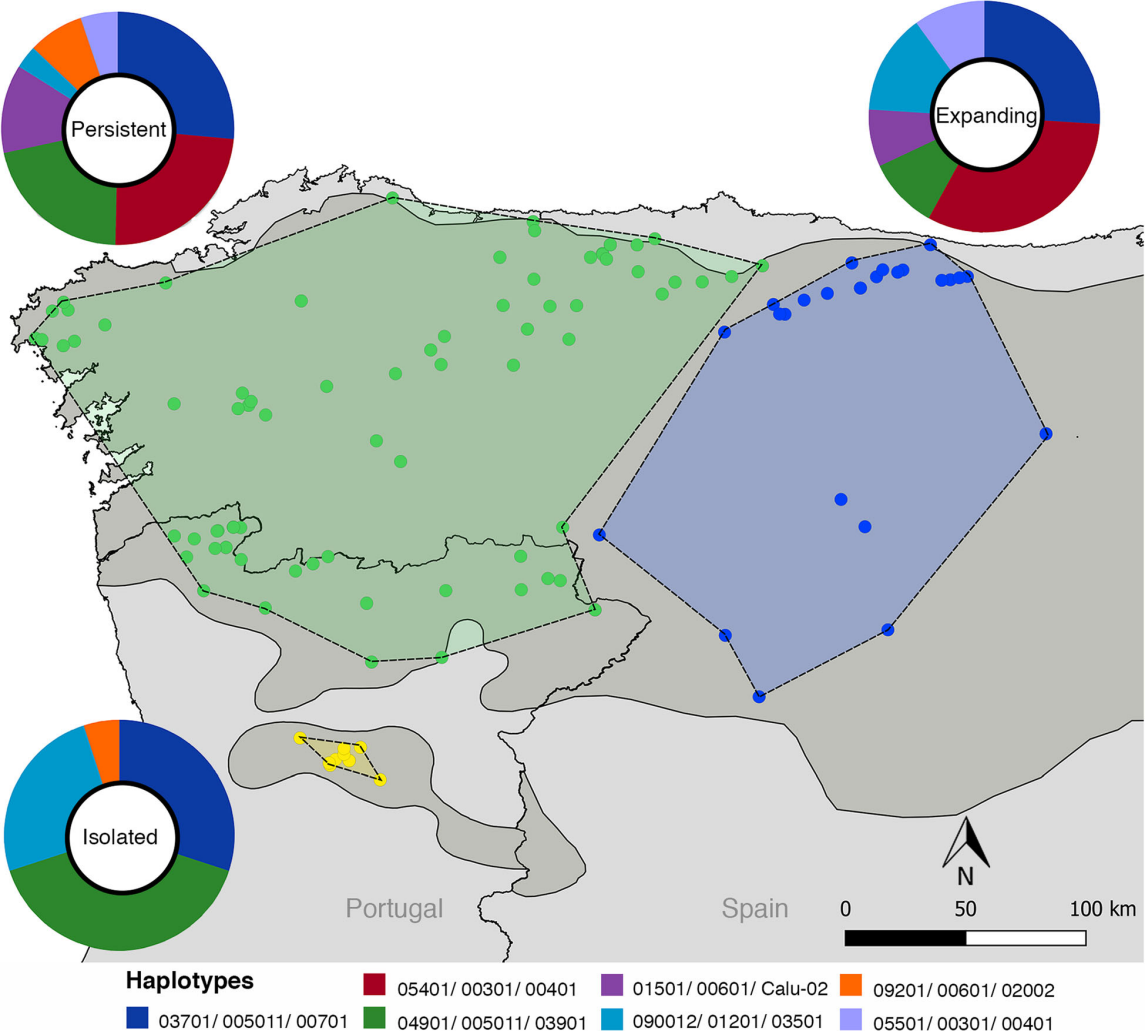
Map showing location of samples corresponding to three demographic groups (persistent in green, expanding in blue and isolated in yellow). Pie charts represent the relative frequency distribution of seven three-locus MHC haplotypes per demographic group. Each color of the pie chart represents a three-locus MHC haplotype, and size is proportional to haplotype frequency within demographic group (Additional file 2: Table S2). Filled gray polygon represents the estimated wolf distribution in the Iberian Peninsula in 2005 [24]

In Europe, abundance and distribution of large carnivores (bear, wolf and lynx) have been dramatically shaped by humans during the past few centuries. Although persecuted and driven to or close to extirpation in several countries, most of the remnant populations have stabilized or even expanded recently [23]. One example is the Iberian wolf (*Canis lupus*), which erstwhile ranged over most of the Iberian Peninsula, but after decades of severe human persecution became confined to small and fragmented populations, representing about 1/5 of the former population range [23–25]. In the 1970s, the Iberian wolf population was estimated to be reduced to ca. 700 individuals, mostly restricted to the northwestern region [23, 26, 27]. After the 1970s, the population started to grow and expanded southwards and eastwards (Fig. 1) [28]. Currently, the Iberian wolf is considered under a stable or positive demographic trend [23]. It occurs in a core area where the species have always persisted in northwestern Iberia [26, 27, 29], and the adjacent re-colonized area (the expansion front), alongside two small and isolated populations [30–32]. Such a complex demographic history have translated into a cryptic population structure in the Iberian wolf at small spatial scales, with moderate level of genetic differentiation, including the differentiation of the re-colonized area [33].

Remarkable MHC class II variability has been shown in wolf populations [34–37]. However, results vary regarding the predominant role of selection over neutral forces. Some studies provided evidence that wolf MHC diversity is maintained by balancing selection, including studies on populations under demographic decline [34, 36], where parasite resistance was suggested as the possible driving force [36]. In contrast, MHC diversity in the bottlenecked wolf population of Scandinavia was shown to be compatible with neutral evolution [38]. Although MHC variability patterns of small and isolated populations may differ from that in large and outbred populations, the power of balancing selection acting on MHC can be outweighed by demographic events, such as bottlenecks and fragmentation with consequent genetic drift [38, 39]. Hence the signature of selection and/or population demographic effects is expected to vary across populations under different demographic histories, challenging the assessment of the relative role of different microevolutionary forces [40].

In this study, we integrate neutral and adaptive diversity patterns to disentangle how adaptive diversity is driven under three different demographic within the Iberian wolf range. Taking into account the genetic population structure of this population based on neutral markers [33], we used the permanent wolf range (i.e., persistent group) as the baseline for demographic stability and compared it to the population dominated by the re-colonization process after the 1970s bottleneck [27] (i.e., expanding group) and to the remaining small and isolated population in the South of Douro River in Portugal (i.e., isolated group). For the persistent group we expect to find natural selection acting to maintain adaptive variation at a stronger intensity than genetic drift; and therefore MHC diversity is expected to exceed neutral diversity. In this scenario, we can either detect balancing or diversifying selection. We may observe balancing selection when MHC exhibits lower population differentiation than neutral loci, due to enhanced effective migration rate and similar allele frequencies in MHC loci [11]. Alternatively, we can detect diversifying selection when MHC exhibits accelerated divergence as a response to spatial or temporal heterogeneity in parasite abundance and diversity [41]. Contrastingly, for expanding and isolated groups, genetic drift is expected to outweigh selection; and therefore similar diversity patterns between MHC and neutral markers are expected.

## Results

### MHC diversity

We found seven DRB1, four DQA1, and six DQB1 alleles across the 113 Iberian wolf samples (Additional file 1: Table S1), all previously reported in other wolf populations (Additional file 2: Table S2, Additional file 3: Table S3). Diversity at DRB1 and DQB1 was higher than diversity at DQA1 (Additional file 4: Table S4). Observed heterozygosity was higher than expected at all three loci considering the whole dataset, with significant departure from Hardy-Weinberg Equilibrium (Additional file 4: Table S4). Nucleotide and amino acid distances were higher for DRB1 and DQB1 than for DQA1, with amino acid distances higher than the nucleotide distances (Additional file 4: Table S4).

When clustering the three loci (DRB1/DQA1/DQB1), we observed seven haplotypes (Fig. 1, Table 1). Each DRB1 allele is found in haplotypic association with a different DQ pair, with exception of alleles DRB1*05401 and DRB1*05501, which share the same DQ pair, DQA1*00301/ DQB1*00401.

**Table 1:** Sequence diversity and neutrality tests of the three demographic groups (persistent, expanding and isolated) and the whole Iberian wolf range for the three-locus (DRB1/DQA1/DQB1) MHC haplotypes

Group	n	h	S	π	η	θ_w_	Tajima’s D	Fu & Li D*
Persistent	78	7	86	0.041	102	0.019	2.580*	2.609**
Expanding	25	6	80	0.040	90	0.022	2.181*	2.098**
Isolated	10	4	79	0.040	87	0.028	1.269	0.330
Iberia	113	7	86	0.041	102	0.018	2.942**	2.751**

Information in the table includes sample size (n), number of haplotypes (h), number of segregating sites (S), nucleotide diversity (π), number of mutations (η), Watterson’s mutation parameter (θ_W_), neutrality tests of Tajima’s D and Fu &Li D*. Statistical significance: *P < 0.05, **P < 0.02

**Fig. 2:**
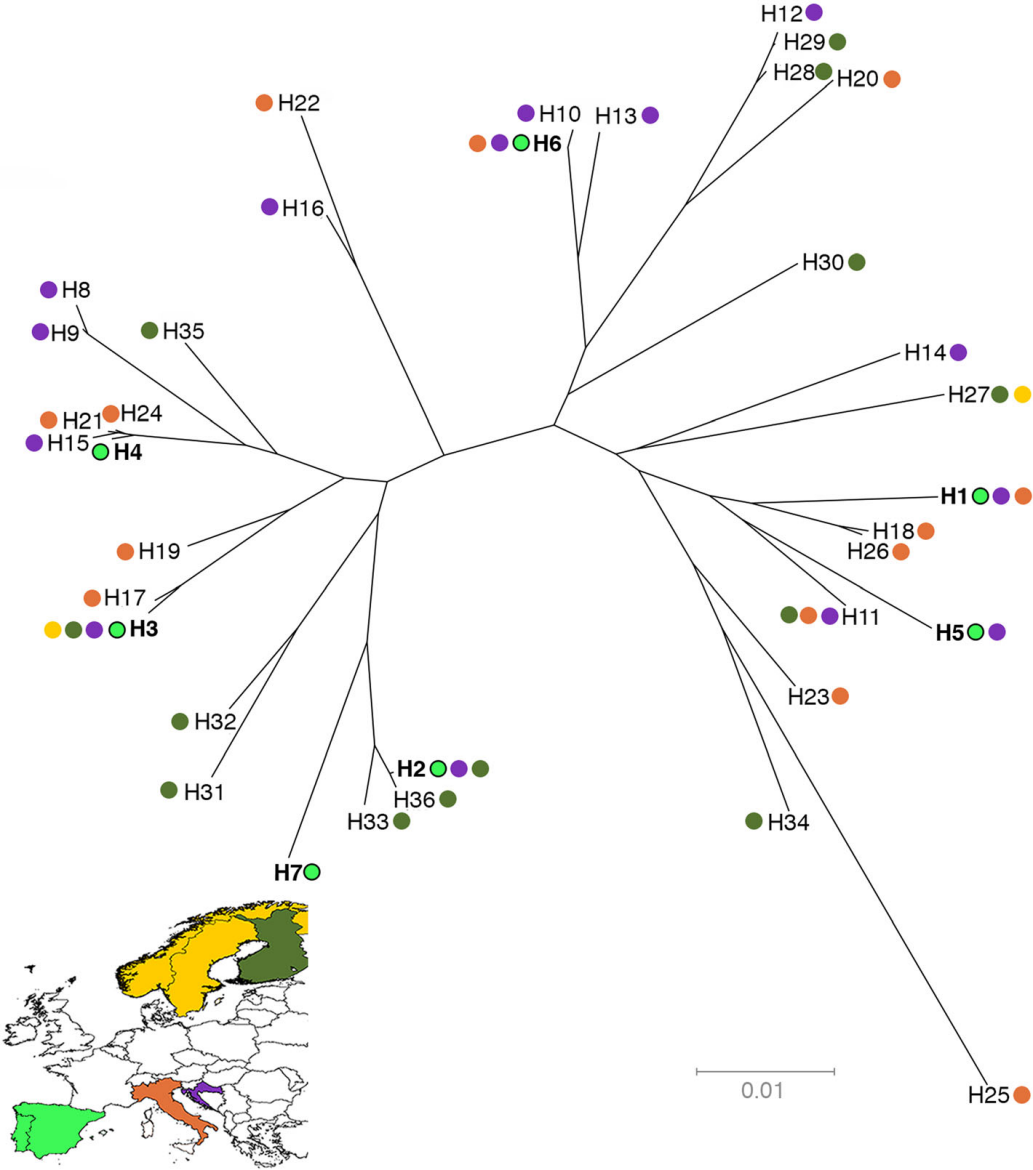
Neighbor joining tree showing relationships between three-locus haplotypes of Iberian and other European wolf populations using p-distances. Colored circles indicate populations where haplotypes were found. Iberian haplotypes are in bold. Haplotype nomenclature is given in Additional file 1: Table S1

Five haplotypes are shared with other European wolf populations (Additional file 3: Table S3). We cannot confirm the presence of two haplotypes in other European wolf populations due to the absence of haplotype reconstruction in [37], where all alleles that compose these two haplotypes were reported. The three most frequent three-locus haplotypes are shared with Croatia, Italy and Finland (Fig. 2, Additional file 2: Table S2, Additional file 3 Table S3). Iberian haplotypes are distributed throughout the NJ tree (Fig. 2). Seven, six and four three-locus haplotypes were found in the persistent, expanding and isolated groups, respectively (Fig. 1, Table 1). The most frequent haplotype was different for each demographic group (Fig. 1, Additional file 2: Table S2). Sequence diversity as the number of segregating sites, nucleotide diversity, number of mutations and Watterson’s mutation parameter were similar among the three different demographic groups (Table 1).

### Positive selection

All MHC loci revealed positive and significant departure from neutrality (Additional file 4: Table S4), indicating an excess of high-frequency segregating sites. Persistent and expanding groups revealed positive and significant departure from neutrality for Tajima’s D and Fu & Li D* tests (Table 1). The isolated group did not show significant departure from neutrality for Tajima’s D and Fu & Li D* tests (Table 1). The dN/dS ratio was significantly different from neutral expectations in all demographic groups both at all sites and at the peptide binding region for DRB1 loci, and only at all sites for DQB1 loci (Table 2). For DQA1, the dN/dS ratio was significantly different from neutral expectations at all sites in isolated group and at the peptide binding region in persistent and expanding group (Table 2).

**Table 2:** Z-tests of selection on all sites, peptide binding region (PBR) inferred from [42], and the non-peptide binding region (non-PBR) of the three demographic groups (persistent, expanding and isolated) of Iberian wolves for the three MHC loci (DRB1, DQA1, and DQB1)

	Persistent	Expanding	Isolated
DRB1			
All	3.339***	3.371***	3.381***
PBR	2.686**	2.780**	2.778**
Non-PBR	1.283	1.548	1.345
DQA1			
All	1.486	1.258	2.090*
PBR	2.192*	2.377*	1.565
Non-PBR	−0.091	−0.230	1.358
DQB1			
All	2.924**	2.625**	2.375*
PBR	1.829	1.742	1.424
Non-PBR	1.644	1.411	1.450

Statistical significance: *P < 0.05, **P < 0.01, ***P < 0.001

Additionally, we found several MHC codons under positive selection in the three demographic groups using OmegaMap, a few having high posterior probability (PP > 95%, Additional file 5: Table S5). DRB1 and DQB1 loci had higher proportions of selected codons than DQA1 (Additional file 5: Table S5). We matched our alignment with the human orthologue [42] and observed that almost all selected codons with high posterior probability (DRB1: 6 in 7, DQA1: 1 in 2, DQB1: 4 in 4 codons) match the same peptide-binding sites described by these authors (Additional file 5: Table S5).

### Genetic differentiation between demographic groups

We found a single model with support from the data to explain allelic richness and expected heterozygosity, with a deviance explained of more than 65% (Additional file 6: Table S6). Allelic richness is explained by a combination of demographic group and locus type, while expected heterozygosity is explained by demographic group, both including the effect of locus (Additional file 6: Table S6). For both the number of alleles and observed heterozygosity we found three models with support, presenting a deviance explained between 20 and 43% (Additional file 6: Table S6). Number of alleles is explained by demographic group or a combination of demographic group and locus type (Additional file 6: Table S6). Observed heterozygosity is explained by the null model or by locus type (Additional file 6: Table S6).

According to the selected models, allelic richness, expected heterozygosity and the number of alleles are significantly higher in the persistent than in the isolated group (Additional file 7: Table S7). Number of alleles is also significantly higher in the persistent than in the expanding group (Additional file 7: Table S7). Allelic richness and observed heterozygosity are significantly higher in MHC than in microsatellites.

### Comparing MHC and neutral diversity

For both MHC and microsatellite loci, significant deviations from Hardy-Weinberg Equilibrium were observed for the persistent and expanding groups (Table 3). However this deviation is in opposite directions (Table 3). The persistent group showed significant higher expected than observed heterozygosity in both molecular markers, while the expanding group showed significant higher observed than expected heterozygosity in MHC (Table 3). Observed heterozygosity was significantly higher at MHC than at microsatellite loci for the persistent and expanding groups (*p* < 0.05, t-test, Additional file 8: Table S8). For the expanding group, the mean allelic richness and expected heterozygosity at MHC were also significantly higher than the ones at microsatellite loci (*p* < 0.05, t-test, Additional file 8: Table S8). For the isolated group, none of the diversity indices was significantly different between MHC and microsatellite loci (*p* > 0.05, t-test, Additional file 8: Table S8).

**Table 3:** Genetic diversity of the three demographic groups (persistent, expanding and isolated) and the whole Iberian wolf range for microsatellite loci and for the three-locus (DRB1/DQA1/DQB1) MHC haplotypes

	Microsatellites							MHC						
Group	n	Na	Ne	AR	Ho	He	F_IS_	N	Na	Ne	AR	Ho	He	F_IS_
Persistent	76	5.5 (0.3)	3.2 (0.2)	3.5	0.578 (0.027)	0.639 (0.028)	0.10* (0.015)	78	5.7 (0.9)	4.1 (0.7)	4.9	0.675 (0.017)	0.742 (0.048)	0.085* (0.039)
Expanding	25	4.2 (0.2)	2.8 (0.1)	3.2	0.561 (0.033)	0.584 (0.03)	0.04* (0.028)	25	5.0 (0.6)	3.7 (0.5)	4.8	0.813 (0.035)	0.724 (0.034)	−0.124* (0.008)
Isolated	9	3.3 (0.2)	2.3 (0.1)	2.9	0.546 (0.36)	0.523 (0.024)	−0.05 (0.047)	10	4.0 (0.6)	2.8 (0.8)	4.0	0.733 (0.167)	0.613 (0.089)	−0.160 (0.131)
Iberia	110	4.3 (0.2)	2.8 (0.1)	3.593	0.562	0.582	0.02	113	4.9 (0.4)	3.6 (0.3)	5.095	0.741 (0.053)	0.693 (0.036)	−0.066 (0.055)

Information in the table includes sample size (n), mean number of alleles per locus (Na), mean number of effective alleles (Ne), mean allelic richness (AR), mean observed and expected heterozygosities (Ho and He), mean inbreeding coefficient (F_IS_). Standard error is given between brackets. Statistical significance: *P < 0.02

**Fig. 3:**
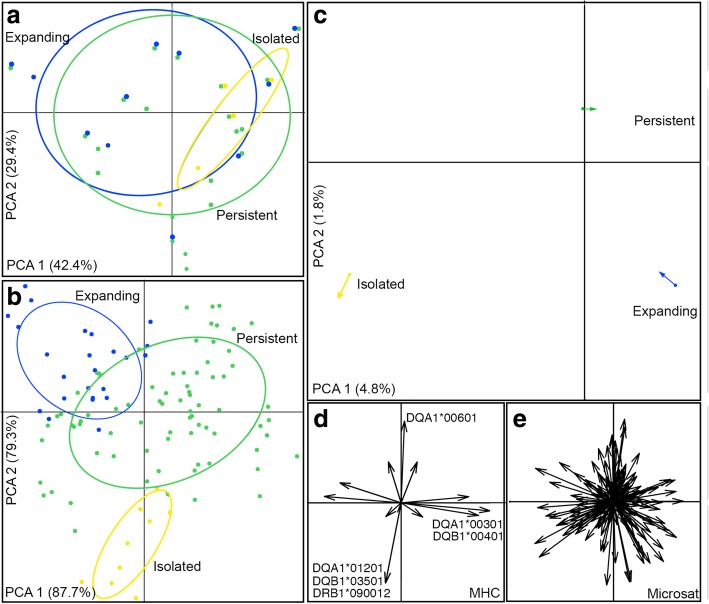
Co-inertia analysis (CoA) between neutral and adaptive data for all demographic groups. **a**. Principal Component Analysis (PCA) for MHC loci; **b**. PCA for microsatellite loci; dots represent different individuals and circles around dots with different colors represent demographic groups. **c**. CoA plot indicating the relative position of each demographic group on the factorial plane for the first two CoA eigenvalues. Dots and arrows represent the projected co-ordinates of each dataset (MHC and microsatellite loci, respectively), which are joined by a vector, where the length of the vector is proportional to the divergence between the datasets. **d**. Canonical weights for MHC alleles. **e**. Canonical weights for microsatellite alleles

The first two components of PCA based on MHC loci did not differentiate among the three demographic groups (Fig. 3). Pairwise F_ST_ values using MHC data support the lack of genetic differentiation between persistent and expanding groups, while moderately significant differentiation is observed between all groups using Jost’s D differentiation (Table 4). Contrastingly, PCA based on microsatellite data showed clear genetic differentiation between all demographic groups, especially for the isolated group, while a partial overlap is observed between persistent and expanding groups (Fig. 3). All F_ST_ and Jost’s D pairwise comparisons between groups were significant for genetic differentiation using microsatellite loci (Table 4). Overall, pairwise genetic differentiation using F_ST_ was lower in MHC than in microsatellite loci, whereas for Jost’s D differentiation index, the isolated group exhibited higher values for MHC than for microsatellite loci (Table 4). The global co-inertia RV coefficient observed was high (0.98), indicating strong correlation between MHC and microsatellite matrices, but this coefficient is not different from what is expected by chance (*p*-value = 0.17).

**Table 4:** Pairwise differentiation values for microsatellite and MHC loci (X/X, respectively), considering three demographic groups of Iberian wolves, estimated for F_ST_ and for Jost’s differentiation index (D)

	F_ST_	D
Persistent/Expanding	0.064**/0.018	0.117**/0.052*
Persistent /Isolated	0.107**/0.084**	0.213**/0.235**
Expanding/Isolated	0.150**/0.128**	0.268**/0.350**

Statistical significance: *P < 0.01, **P < 0.001

## Discussion

This is the first report of MHC class II diversity in the Iberian wolf. Overall, Iberian wolves exhibit lower MHC diversity than their European counterparts, both in number of alleles and number of three-locus haplotypes (i.e., 6–7 alleles and 7 haplotypes in Iberia vs 6–13 alleles and 13–14 haplotypes in other European populations) [34–36]. The exception is the isolated Scandinavian wolf population, which went through a drastic bottleneck recovering from only three individuals [38]. The reduced MHC diversity in the Iberian wolf has also been observed for other genomic regions [31, 43–45]. Long-term isolation and past bottlenecks in the Iberian population [27, 45] have certainly reduced neutral diversity and may also be at the origin of this depleted MHC diversity.

All MHC alleles have been previously identified in other canids [37, 46, 47], a pattern that is better explained by the trans-species polymorphism described in MHC canid phylogenies [37, 48] than by hybridization which is not common in the Iberian wolf [49]. The observed 17 alleles were confined in just seven three-locus haplotypes of which five have previously been reported [34–36, 48]. The remaining two haplotypes were not unequivocal confirmed to be present in other wolf populations due to the absence of haplotype reconstruction in [37]. The occurrence of conserved three-locus MHC haplotypes in Iberian wolves confirms the tight linkage of these gene loci, supporting a strong selective pressure maintaining haplotype combinations. The association observed between each DRB1 allele and a specific DQ pair supports the preferential association between alleles at these loci [46]. A similar trend is observed in other European wolf population [34–36], though not as extreme as in Iberia, where seven DRB1 alleles yielded only seven haplotypes. However, similarly to other European populations, the seven haplotypes are widely distributed in the NJ tree, supporting maximal MHC diversity in the Iberian wolf.

Within Iberia, we found contrasting MHC diversity patterns according to different demographic trajectories. The persistent group exhibited significant higher genetic diversity than the isolated group, but did not show significant excess of MHC heterozygotes. However, we found evidence of balancing selection acting to maintain MHC adaptive variation in this group, compensating the effects of the severe population decline in the 19th and 20th centuries [50, 51]. This is revealed by a significant departure from neutrality at MHC loci, significant higher observed and expected heterozygosity and lower differentiation at MHC than neutral loci, and signs of positive selection with higher proportions of selected codons lying in peptide-binding regions, as inferred from humans. Similar results have been reported for Finnish wolves where, despite a strong genetic signal of population decline [52], the population still reveals a predominant role of balancing selection maintaining MHC diversity [36]. Although selective advantage has been associated with a heterozygote advantage mechanism, MHC diversity can also be promoted by negative frequency-dependent selection [2]. This model proposes that the emergence of a novel pathogen can promote the frequency of a rare or novel allele/haplotype [2, 3, 53]. The frequency shift of an adaptive allele/haplotype by such process can occur in very few generations [53]. We can therefore not exclude that negative frequency-dependent selection may have contributed to maintain high allelic richness possibly compensating for the low number of heterozygotes observed.

The expanding group exhibited similar genetic diversity than the persistent group but show significant excess of MHC heterozygotes. Reduced genetic diversity is expected for marginal populations [10, 54–56], but increased heterozygosity at MHC supports a model of heterozygote advantage for this demographic group. The positive and significant result for neutrality statistics provides further evidence for balancing selection acting in this demographic group. The overdominance model postulates that heterozygotes have higher fitness than homozygotes due to the wider spectrum of MHC receptors able to induce parasite resistance [2]. MHC heterozygotes have indeed been associated with resistance to several infections in wolves [36]. Thus, the high MHC genetic diversity exhibited by the expanding group is expected to maintain its high adaptive potential.

The isolated group showed the lowest neutral and adaptive diversity and no significant excess of MHC heterozygotes, a likely result of the dramatic population decline that culminated in isolation in the early twenty-first century [26, 30]. The observed depletion of MHC diversity potentially increases population susceptibility to disease, and adds to current concern for its survival [12, 57]. Indeed, in a wolf serologic survey the isolated group showed no canine parvovirus antibodies, possibly related to high case-fatality rate [58]. No significant departure from neutrality neither significant difference between MHC and neutral loci were observed, suggesting that MHC loci behave according to neutral expectations in the isolated group. Other examples are known for small and isolated populations where genetic drift outweighs the strength of selection [3, 38, 39]. However, one interesting result comparing the isolated group with the others may suggest selective forces acting in this group. The higher Jost’s D differentiation index observed for MHC relative to neutral loci may be explained by diversifying selection as consequence of spatial variation in pathogen-mediated responses [40] and/or due to stronger divergence due to short-term positive selection on beneficial alleles. Interestingly, the isolated wolf population shares several pathogens with wild and domestic species [59–61], but holds also new pathogens [60], which could be associated with the observed differentiation in MHC. Inconclusive results are probably due to the small sample size of this group and should be interpreted with caution [62].

## Conclusion

By empirically testing adaptive diversity under three different demographic scenarios within the Iberian wolf range, we were able to show that these different demographic scenarios are associated with different selective pressures, which are possibly driven by different selection mechanisms to maintain adaptive diversity. Future studies are warranted to investigate the link between the Iberian wolf immunogenetic diversity and the pathogen community structure to better understand the mechanisms underlying adaptation in each demographic group.

## Methods

### Sampling and DNA extraction

A total of 113 wolf tissue samples were collected across the range of wolves in the Iberian Peninsula in the last two decades. Tissue samples were collected from several Portuguese and Spanish entities (please see Ethics section). To avoid close familiar relationships and the presence of hybrids between wolf and dog in our dataset we selected samples according criteria defined by [33]. Samples were classified into one of three demographic groups: i) persistent group (*n* = 78), represented by samples from the northwest Iberian Peninsula where the wolf has been continuously present in the last decades, ii) expanding group (*n* = 25), represented by samples originally from the area where wolves were absent during the recent low of the population in the 1970s, representing a well-defined genetic cluster within Iberia (Castilla y León cluster) [33], and iii) isolated group (*n* = 10), represented by samples from the small and isolated wolf area south of the Douro river in Portugal (Fig. 1). Samples were stored in ethanol and DNA was further extracted using QIAGEN DNeasy Blood & Tissue Kit.

### MHC amplification and sequencing

DLA class II genes DRB1, DQA1 and DQB1 were amplified and sequenced using intronic and locus specific primers described in [34]. Polymerase chain reactions (PCR) were carried out in a total volume of 10 μl containing approximately 10 ng of DNA, 0.4 μM of each primer and 5 μl 2× MyTaq HS Mix (Bioline) (see Additional file 9: Table S9 for PCR conditions). PCR products were sequenced in both directions using BigDye® Terminator v3.1 Cycle Sequencing Kit (Applied Biosystems) according to the manufacturer’s protocol and sequencing products were separated on a 3130xl Genetic Analyzer (Applied Biosystems).

### MHC alleles and haplotype assignment

DRB1, DQA1 and DQB1 loci sequences of each of the 113 samples were analyzed and aligned with CodonCode Aligner (CodonCode Corporation, www.codoncode.com) and BioEdit [63].

MHC sequences were phased in DnaSP v. 5.10 [64] using PHASE [65] with the “recombination” model (−MR0) and 1000 iterations after 100 burn-ins. The threshold for accepting true alleles was set to 90%. This methodology has been shown to be accurate for MHC haplotype reconstruction [66], retrieving very low false positives [67]. MHC alleles obtained for each locus were compared with available information in public datasets (NCBI: https://www.ncbi.nlm.nih.gov) for the genus *Canis*. As the three genes studied are chromosomally clustered in tandem, we analyzed each locus individually or as three-locus haplotypes. Three-locus haplotypes (DRB1/DQA1/DQB1) were reconstructed firstly in individuals homozygous for each locus, and then for heterozygous individuals basing inferences on haplotypes observed in homozygous state. Haplotype reconstruction was confirmed with available information for other canid populations.

### MHC diversity

MHC diversity was analysed for each locus and for the three-locus haplotypes. For both we estimated number of alleles (NA), mean number of effective alleles (Ne), mean observed (Ho) and expected (He) heterozygosities and inbreeding coefficient (F_IS_) using GENALEX 6.5 [68]. Test of deviation from Hardy-Weinberg equilibrium was calculated using GENEPOP 4.6 [69]. Allelic richness (AR) was estimated using FSTAT 2.9.3.2 [70], which performs rarefaction compensating sampling disparity, reducing all populations to a minimum sample size of *n* = 10. Sequence-level diversity values, such as number of haplotypes (h), number of segregating sites (S), nucleotide (π) and haplotype (HD) diversities, and Watterson’s mutation parameter (θw) were estimated either in each locus or each group (with the three-locus haplotypes) using DnaSP 5.10.

Molecular divergence analysis over all sequence pairs, also either in each locus or each group (with the three-locus haplotypes) was conducted using Mega 7 [71] with a 1000 replicate bootstrap procedure. The best nucleotide and amino acid substitution models were chosen according to the Bayesian Information Criterion and used to compute nucleotide and amino acid evolutionary distances by the maximum-likelihood method through 1000 bootstrap replicates. The relationship between three-locus haplotypes of Iberian and other European wolf populations was reconstructed using the Neighbor Joining (NJ) method and p-distances in Mega 7. Three-locus haplotypes of other European wolf populations were retrieved from previous studies [34–36, 38].

### Testing positive selection

To detect evidence of selection on MHC loci at the population-level, we estimated Tajima’s D [72] and Fu & Li D* [73] statistics using DnaSP 5.10. Under neutrality, D and D* are not different from 0, and significant negative deviations may indicate a recent selective sweep or population expansion while positive deviations indicate balancing selection or population contraction. Neutrality tests were computed for each MHC locus and for the three-locus haplotypes in the whole Iberian wolf range and in each demographic group. Deviations from neutrality were also assessed by a Z-test (non-synonymous substitution ≠ synonymous substitution; dN ≠ dS) implemented in MEGA 7, using Nei-Gojobori method with Jukes-Cantor correction for multiple substitutions and 1000 bootstrap replicates. Z-tests were computed for all sites, antigen-binding sites inferred from the human orthologue [42], and non-antigen binding sites for each demographic group. Additionally, we also used a “coalescent with recombination” Bayesian approximation implemented in OmegaMap [74] to find signatures of selection in each demographic group. OmegaMap allows both selection parameter (ω) and recombination rate (ρ) to vary along the sequence [75]. Codons with excess of non-synonymous polymorphism compared to synonymous polymorphism are assumed under positive selection. We followed [36] to set distribution and parameter values of priors to represent neutrality. We performed two runs for each locus and each demographic group with 250,000 repeats and a burn-in of 20,000. Convergence of runs was check through summary function implemented in R 3.5.0 (R Core Team, 2018). Codons are considered as positively selected with posterior probabilities (PP) above 95%.

### Microsatellite diversity

We selected a set of 39 microsatellite loci previously genotyped by [33] for all but three samples (two from the persistent and one from the isolated groups) sequenced for MHC to estimate neutral genetic diversity in each demographic group. This set included only loci with less than 25% missing data and with no significant departure from Hardy–Weinberg equilibrium after Bonferroni correction estimated using GENALEX 6.5. Genetic diversity, including number of alleles (NA), mean number of effective alleles (Ne), mean allelic richness (AR), mean observed (Ho) and expected (He) heterozygosities and inbreeding coefficient (F_IS_) were estimated using GENALEX 6.5 and FSTAT 2.9.3.2. For allelic richness all populations were reduced to a minimum sample size of *n* = 5. Test of deviation from Hardy-Weinberg equilibrium was calculated using GENEPOP 4.6.

### Testing for genetic differentiation between demographic groups

To test for statistically significant differences of genetic diversity between the demographic groups, we performed linear mixed effects models (e.g.,[76, 77]) using *lme4* [78] and *lmerTest* [79] implemented in R platform (R Core Team, 2018). We used number of alleles, allelic richness, observed and expected heterozygosities as dependent variables, demographic group and locus type (MHC and microsatellites) as explanatory factors, and locus as random variable. Ten competing models were tested, including i) null, ii) demographic group-dependent, iii) locus type-dependent, iv) dependent on both demographic group and locus type, and v) dependent on both demographic group and locus type plus the association between these two factors. For each of these options we run two models accounting or not for locus random effect (for model syntaxes see Additional file 6: Table S6). We performed independent model testing for each diversity measure. We used Akaike Information Criterion corrected for small sample sizes, AICc, to determine the best model. Models with delta AICc (ΔAICc) lower than 2.0 were assumed as equally plausible. For each diversity measure we present estimates, standard errors and probability inferred from t-value, indicating the precision of the estimates.

### Comparing neutral and functional diversity

Expectations that functional diversity exceeds neutral diversity were tested by a one-tailed t-test (*p*-value), following [35]. The relationship between microsatellite and MHC loci was estimated using a co-inertia analysis (CoA) following [10]. CoA is a multivariate method that estimates the covariance structure between two tables with the same observations (e.g., same individuals). For each genetic matrix (microsatellite and MHC loci) we performed a Principal Component Analysis (PCA) followed by factorial PCA using demographic groups as explanatory variable. For this analysis we used the *adegenet* and *ade4* R packages [80, 81]. We then performed a CoA using the two most important PCA components. In a CoA plot, an arrow and a dot define each demographic group, where the length of the vector connecting arrow and dot is proportional to the divergence between the types of markers. The correlation coefficient RV was used to evaluate the strength of the relationship between the two matrices. Values of RV close to 1 indicate strong correlation between matrices. The significance of the RV coefficient was tested using 1000 permutations, where null hypothesis is that the two matrices are related by chance. Canonical weights for microsatellite and MHC alleles were used to disclose the contribution of each allele to the divergence of demographic groups.

MHC and neutral differentiation between groups was estimated through F_ST_ [82] and Jost’s D [83] using Arlequin 3.5.2.2 [84] and the DEMEtics R package [85], respectively. F_ST_ and Jost’s D differentiation measures were both used because F_ST_ may be affected by highly variable markers [39].

## Additional files

Additional file 1: **Table S1.** DLA-DRB1, DLA-DQA1 and DLA-DQB1 alleles and their frequency (f) in the three assigned demographic groups and whole Iberian wolf range. (PDF 19 kb)

Additional file 2: **Table S2.** Number of three-locus (DLA-DRB1/DQA1/DQB1) haplotypes (N) and their frequency (f) in the three assigned demographic groups and whole Iberian wolf range. (PDF 14 kb)

Additional file 3: **Table S3.** Comparison of the DLA-DRB1, DQA1, DQB1 alleles and three-locus haplotypes (DRB1/DQA1/DQB1) in the Iberian (this study), Croatian [34], Italian [35], Finnish [36] wolf populations. Underlined: shared alleles and haplotypes between Iberia and at least one of the three other European wolf populations; *alleles and haplotypes found in other European wolf populations [37, 38] ^¥^alleles found in the North American wolf population [37, 48]; # haplotypes that are possibly found in other European wolf populations, three-locus haplotypes were not reconstruct but all alleles are present [37]. NI: no DRB1 allele detected. (PDF 162 kb)

Additional file 4: **Table S4.** Genetic diversity values for the three MHC loci in the Iberian wolf range. Sample size (n), mean number of alleles (Na), mean number of effective alleles (Ne), mean observed and expected heterozygosities (Ho and He), mean inbreeding coefficient (F_IS_), number of segregating sites (S), nucleotide diversity (π), number of mutations (η) Watterson’s mutation parameter (θ_W_), neutrality tests of Tajima’s D and Fu &Li D*, and estimates of average nucleotide and aminoacid distances. Statistical significance: *P < 0.05, **P < 0.02. Standard error estimates shown in brackets. (PDF 139 kb)

Additional file 5: **Table S5.** Positively selected codons in each Iberian wolf demographic group according to OmegaMap analysis and codons putatively belonging to the peptide-binding regions (PBR) suggested by [42] for DLA-DRB1, DLA-DQA1 and DLA-DQB1. Bold and underlined positions denote codons identified to be under positive selection with posterior probability > 0.95. (PDF 110 kb)

Additional file 6: **Table S6.** Model selection information, including Akaike Information Criterion (AICc), delta AICc (ΔAICc) and weight of selected model for number of alleles (Na), allelic richness (AR), observed (Ho) and expected (He) heterozygosities. (PDF 128 kb)

Additional file 7: **Table S7.** Estimates of combined diversity measures of each demographic group, standard errors (S.E.) and probability inferred from t-value, according to the selected model. (PDF 11 kb)

Additional file 8: **Table S8.** Mean number of alleles (Na), mean number of effective alleles (Ne), mean allelic richness (AR), observed (Ho) and expected (He) heterozygosities, and mean inbreeding coefficient (F_IS_) at the microsatellite and MHC loci, in three demographic groups (persistent, expanding and isolated), including standard deviation (s.d). The probability of the values to differ between microsatellite and MHC loci was tested by a one-tailed t-test (*p*-value). Values with statistical significance are in bold (*p* < 0.05). (PDF 28 kb)

Additional file 9: **Table S9.** Thermocycling profile for PCR amplification of the three MHC fragments used in this work. Annealing temperature (AT) was set initially at 64 °C (DRB1), 54 °C (DQA1) and 76 °C (DQB1). (PDF 97 kb)

## Abbreviations

AICc: Akaike Information Criterion corrected for small sample sizes
AR: Mean allelic richness
CoA: Co-inertia analysis
dN: Non-synonymous substitution
dS: Synonymous substitution
F_IS_: Inbreeding coefficient
h: number of haplotypes
HD: Haplotype diversity
He: Expected heterozygosity
Ho: Observed heterozygosity
MHC: Major histocompatibility complex
NA: Number of alleles
Ne: Mean number of effective alleles
NJ: Neighbor Joining
PCA: Principal Component Analysis
PCR: Polymerase chain reaction
PP: Posterior probability
RV: Correlation coefficient
S: Number of segregating sites
ΔAICc: Delta AICc
θw: Watterson’s mutation parameter
π: Nucleotide diversity
ρ: Recombination rate
ω: Selection parameter
