# Supplementary material for: Alternated selection mechanisms maintain adaptive diversity in different demographic scenarios of a large carnivore
Source: BMC Evol Biol. 2019 Apr 11;19:90. doi: 10.1186/s12862-019-1420-5 (PMC6460805; doi:10.1186/s12862-019-1420-5)
Supplement: Supplementary file 1 — Table S1. DLA-DRB1, DLA-DQA1 and DLA-DQB1 alleles and their frequency (f) in the three assigned demographic groups and whole Iberian wolf range. (PDF 19 kb) [file 12862_2019_1420_MOESM1_ESM.pdf]

Additional file 1

Table S1 DLA-DRB1, DLA-DQA1 and DLA-DQB1 alleles and their frequency (f) in the three assigned demographic groups and whole Iberian wolf range.

Locus	Gene nomenclature	Accession number	Allele frequency			
			Iberian	Persistent	Expanding	Isolated
DRB1	03701	AF343738	0.258	0.263	0.260	0.300
	05401	AY126658	0.242	0.244	0.320	-
	04901	AY126655	0.212	0.212	0.100	0.400
	01501 (Cala-DRB1*17)	AF516924	0.106	0.122	0.080	0.000
	090012 (Calu-DRB1*16)	AY126663	0.072	0.032	0.140	0.250
	09201 (Calu-10)	AM408904	0.055	0.077	-	0.050
	05501	AY126662	0.055	0.051	0.100	-
DQA1	005011 (DQA 3)	U44787	0.470	0.474	0.360	0.700
	00301	Y07944	0.297	0.295	0.420	-
	00601 (DQA 6)	U44790	0.161	0.199	0.080	0.050
	01201	AF343734	0.072	0.032	0.140	0.250
DQB1	00401 (DQB 5)	AF043150	0.297	0.301	0.420	0.050
	00701(DQB 4)	AF043149	0.258	0.256	0.260	0.250
	03901	AJ316222	0.212	0.212	0.100	0.400
	Calu-DQB*02	AY126653	0.106	0.122	0.080	-
	03501	AJ31107	0.072	0.032	0.140	0.250
	02002 (DQB 19)	AF043164	0.055	0.077	0.000	0.050
