# Supplementary material for: Alternated selection mechanisms maintain adaptive diversity in different demographic scenarios of a large carnivore
Source: BMC Evol Biol. 2019 Apr 11;19:90. doi: 10.1186/s12862-019-1420-5 (PMC6460805; doi:10.1186/s12862-019-1420-5)
Supplement: Supplementary file 2 — Table S2. Number of three-locus (DLA-DRB1/DQA1/DQB1) haplotypes (N) and their frequency (f) in the three assigned demographic groups and whole Iberian wolf range. (PDF 14 kb) [file 12862_2019_1420_MOESM2_ESM.pdf]

Additional file 2

Table S2 Number of three-locus (DLA-DRB1/DQA1/DQB1) haplotypes (N) and their frequency (f) in the three assigned demographic groups and whole Iberian wolf range.

Haplotype nomenclature	Persistent Group		Expanding Group		Isolated Group		Iberian Population	
**DLA-DRB1/ DQA1/ DQB1**	**N**	**f**	**N**	**f**	**N**	**f**	**N**	**f**
DRB1*03701/ DQA1*005011/ DQB1*00701	41	0.265	13	0.260	6	0.300	41	0.265
DRB1*05401/ DQA1*00301/ DQB1*00401	37	0.239	16	0.320	0	0.000	37	0.239
DRB1*04901/ DQA1*005011/ DQB1*03901	33	0.213	5	0.100	8	0.400	33	0.213
DRB1*01501/ DQA1*00601/ Calu-DQB*02	19	0.123	4	0.080	0	0.000	19	0.123
DRB1*090012/ DQA1*01201/ DQB1*03501	5	0.032	7	0.140	5	0.250	5	0.032
DRB1*09201/ DQA1*00601/ DQB1*02002	12	0.077	0	0.000	1	0.050	12	0.077
DRB1*05501/ DQA1*00301/ DQB1*00401	8	0.052	5	0.100	0	0.000	8	0.052
