# Supplementary material for: Alternated selection mechanisms maintain adaptive diversity in different demographic scenarios of a large carnivore
Source: BMC Evol Biol. 2019 Apr 11;19:90. doi: 10.1186/s12862-019-1420-5 (PMC6460805; doi:10.1186/s12862-019-1420-5)
Supplement: Supplementary file 3 — Table S3. Comparison of the DLA-DRB1, DQA1, DQB1 alleles and three-locus haplotypes (DRB1/DQA1/DQB1) in the Iberian (this study), Croatian [34], Italian [35], Finnish [36] wolf populations. Underlined: shared alleles and haplotypes between Iberia and at least one of the three other European wolf populations; *alleles and haplotypes found in other European wolf populations [37, 38] ¥alleles found in the North American wolf population [37, 48]; # haplotypes that are possibly found in other European wolf populations, three-locus haplotypes were not reconstruct but all alleles are present [37]. NI: no DRB1 allele detected. (PDF 162 kb) [file 12862_2019_1420_MOESM3_ESM.pdf]

Additional file 3

Table S3 Comparison of the DLA-DRB1, DQA1, DQB1 alleles and three-locus haplotypes (DRB1/DQA1/DQB1) in the Iberian (this study), Croatian [1], Italian [2], Finnish [3] wolf populations. Underlined: shared alleles and haplotypes between Iberia and at least one of the three other European wolf populations; *alleles and haplotypes found in other European wolf populations [4, 5] ¥alleles found in the North American wolf population [4, 6]; # haplotypes that are possibly found in other European wolf populations, three-locus haplotypes were not reconstruct but all alleles are present [4]. NI: no DRB1 allele detected.

	Iberian Peninsula	Croatia	Italy	Finland
**DRB1**	03701; 05401*; 04901*; 01501*; 090012*; 09201¥; 05501*	03701; 04301; 04302; 05401; 092011; 092012; 03601; 03202; 01501; 04901; 01502; 01801; 090012	12801; 092013; 03601; 02001; 03202; 01501; 03701; 00101; 092011 (09201)	03601; 03101; 05401; 10001; 04901; 05301; 10101; 05601; 02002; 09901; 03801
**DQA1**	005011*¥; 00301*¥; 00601*¥; 01201*¥	005011; 00301; 00601; 012011; 00201; 00901; 00101	005011; 012011 (01201); 00401; 00201; 00601; 00101	012011; 01101; 00301; 00201; 005011; 014012; 01001
**DQB1**	00401¥; 00701*¥; 03901*; Calu-DQB*02*; 03501*¥; 02002¥	00701; 03801; 00401; 02002; 03501; 04101; 02901; 00101; 03901; 02301; 00802	03901; 00701; 03501; 01303; 02901; 00301; 00201; 02002	03501; 04001; 00401; 02901; 03901; 04401; 01303; 05601; CL246
**DRB1/DQA1/DQ[illegible]**	H1: 03701/005011/00701<br>H2: 05401/00301/00401<br>H3: 04901/005011/03901*<br>H4: 01501/00601/ Calu-DQB*02#<br>H5: 090012/01201/03501<br>H6: 09201/00601/02002<br>H7: 05501/00301/00401#	H1: 03701/005011/00701<br>H8: 04301 or H9: 04302/00301/03801<br>H2: 05401/00301/00401<br>H6: 092011 or H10: 092012/00601/02002<br>H11: 03601/012011/03501<br>H12: 03202/00201/02901<br>H13: 05401/00601/02002<br>H14: 01501/00901/00101<br>H3: 04901/005011/03901<br>H15: 01502/00601/02301<br>H16: 01801/00101/00802<br>H5: 090012/012011/03501	H17: 12801/005011/03901<br>H18: 092013/005011/00701<br>H11: 03601/012011/03501<br>H19: 02001/00401/01303<br>H20: 03202/00201/02002<br>H1: 03701/005011/00701<br>H21: 01501/00601/00301<br>H22: 00101/00101/00201<br>H6: 092011/00601/02002<br>H23: 12801/005011/00701<br>H24: 01501/00401/00301<br>H25: 02001/00401/03901<br>H26: 092013/005011/03501	H11: 03601/012011/03501<br>H27: 03101/01101/04001<br>H2: 05401/00301/00401<br>H28: 10001/00201/02901<br>H3: 04901/005011/03901<br>H29: 10101/00201/02901<br>H30: 05601/014012/04401<br>H31: 05301/00301/00401<br>H32: 05301/01101/ 01303<br>H33: 09901/00301/00401<br>H34: 02002/01001/05601<br>H35: 03801/005011/01303<br>H36: 05401/00301/CL246

References

1. Arbanasic H, Huber D, Kusak J, Gomercic T, Hrenovic J, Galov A. Extensive polymorphism and evidence of selection pressure on major histocompatibility complex DLA-DRB1, DQA1 and DQB1 class II genes in Croatian grey wolves. Tissue Antigens. 2013;81:19–27.

2. Galaverni M, Caniglia R, Fabbri E, Lapalombella S, Randi E. MHC variability in an isolated wolf population in Italy. J Hered. 2013;104:601–12.

3. Niskanen AK, Kennedy LJ, Ruokonen M, Kojola I, Lohi H, Isomursu M, et al. Balancing selection and heterozygote advantage in major histocompatibility complex loci of the bottlenecked Finnish wolf population. Mol Ecol. 2014;23:875–89.

4. Seddon JM, Ellegren H. MHC class II genes in European wolves: A comparison with dogs. Immunogenetics. 2002;54:490–500.

5. Seddon JM, Ellegren H. A temporal analysis shows major histocompatibility complex loci in the Scandinavian wolf population are consistent with neutral evolution. Proc R Soc B Biol Sci. 2004;271:2283–91. doi:10.1098/rspb.2004.2869.

6. Kennedy LJ, Angles JM, Barnes A, Carmichael LE, Radford AD, Ollier WER, et al. DLA-DRB1, DQA1, and DQB1 alleles and haplotypes in North American gray wolves. J Hered. 2007;98:491–9.
