# Supplementary material for: Alternated selection mechanisms maintain adaptive diversity in different demographic scenarios of a large carnivore
Source: BMC Evol Biol. 2019 Apr 11;19:90. doi: 10.1186/s12862-019-1420-5 (PMC6460805; doi:10.1186/s12862-019-1420-5)
Supplement: Supplementary file 4 — Table S4. Genetic diversity values for the three MHC loci in the Iberian wolf range. Sample size (n), mean number of alleles (Na), mean number of effective alleles (Ne), mean observed and expected heterozygosities (Ho and He), mean inbreeding coefficient (FIS), number of segregating sites (S), nucleotide diversity (π), number of mutations (η) Watterson’s mutation parameter (θW), neutrality tests of Tajima’s D and Fu &Li D*, and estimates of average nucleotide and aminoacid distances. Statistical significance: *P < 0.05, **P < 0.02. Standard error estimates shown in brackets. (PDF 139 kb) [file 12862_2019_1420_MOESM4_ESM.pdf]

Additional file 4

Table S4 Genetic diversity values for the three MHC loci in the Iberian wolf range. Sample size (n), mean number of alleles (Na), mean number of effective alleles (Ne), mean observed and expected heterozygosities (Ho and He), mean inbreeding coefficient (F_{IS}), number of segregating sites (S), nucleotide diversity (π), number of mutations (η) Watterson´s mutation parameter (θ_W), neutrality tests of Tajima's D and Fu &Li D*, and estimates of average nucleotide and aminoacid distances. Statistical significance: *P< 0.05, **P<0.02. Standard error estimates shown in brackets.

Locus	n	Na	Ne	Ho	He	F_{IS}	S	π	η	θ_w	Tajima's D	Fu & Li D*	Nucleotide distance[1]	Aminoacid distance[2]
**DRB1**	113	6.0	4.3	0.826	0.707	-0.081*	42	0.052	51	0.028	2.572*	2.356**	0.235 (0.119)	0.338 (0.137)
**DQA1**	113	4.0	2.6	0.627	0.601	-0.034*	10	0.016	10	0.007	3.061**	1.321	0.022 (0.007)	0.055 (0.021)
**DQB1**	113	5.3	5.3	0.809	0.747	-0.087*	34	0.053	41	0.027	2.810**	2.217**	0.398 (0.278)	0.272 (0.098)

[1]Analyses were conducted using the T92+G (Tamura 3-parameter) model for DRB1 locus, JC model for DQA1 locus and JC+G for DQB1 locus. [2]Analyses were conducted using the JTT for DQA1 locus and JTT+G for DRB1 and DQB1 loci. Analysis included 7, 4 and 6 sequences corresponding to the found DRB1, DQA1 and DQB1 alleles, respectively.
