# Supplementary material for: Alternated selection mechanisms maintain adaptive diversity in different demographic scenarios of a large carnivore
Source: BMC Evol Biol. 2019 Apr 11;19:90. doi: 10.1186/s12862-019-1420-5 (PMC6460805; doi:10.1186/s12862-019-1420-5)
Supplement: Supplementary file 5 — Table S5. Positively selected codons in each Iberian wolf demographic group according to OmegaMap analysis and codons putatively belonging to the peptide-binding regions (PBR) suggested by [42] for DLA-DRB1, DLA-DQA1 and DLA-DQB1. Bold and underlined positions denote codons identified to be under positive selection with posterior probability > 0.95. (PDF 110 kb) [file 12862_2019_1420_MOESM5_ESM.pdf]

Additional file 5

Table S5 Positively selected codons in each Iberian wolf demographic group according to OmegaMap analysis and codons putatively belonging to the peptide-binding regions (PBR) suggested by [1] for DLA-DRB1, DLA-DQA1 and DLA-DQB1. Bold and underlined positions denote codons identified to be under positive selection with posterior probability > 0.95.

	Positively selected codons in OmegaMap																							
**DRB1**	7	8	9	11	14	24	25	26	28	30	35	36	45	55	58	61	65	68	69	72	76	82	84	88
**PBR**	*		*	*				*	*		*	*		*	*		*	*	*	*	*		*	
Persistent (n=78)	X	X	**X**	X	X	**X**	X	**X**	**X**	X	**X**	X	X	**X**	X	X		X	X	X	X	X	X	
Expanding (n=25)	X	X	**X**	X	X	**X**	X	**X**	**X**	X	**X**	X	X	**X**	X	X	X	X	X	X	X	X	X	X
Isolated (n=10)		X	**X**	X		X	X	X	X	X	X	X	X	X	X	X	X	X	X	X	**X**	X	X	X
**DQA1**	20	50	63	64	71	74	77																	
**PBR**	*		*	*	*	*																		
Persistent (n=78)	X	X	X	**X**	X	X	X																	
Expanding (n=25)	X	**X**	X	X	X	X	X																	
Isolated (n=10)	X	**X**		X	X		X																	
**DQB1**	4	8	23	24	25	32	42	52	58	61	62	63	64	65	66	69	70	72	80	84				
**PBR**	*	*	*		*	*		*			*			*	*	*			*					
Persistent (n=78)	X	**X**	X	X	X	X	X	**X**	X	X	**X**	X	X	X	**X**	X	X	X	X	X				
Expanding (n=25)	X	X	X	X	X	X		**X**	X	X	X	X	X	X	X				X	X				
Isolated (n=10)	X	X	X	X	X	X	X	**X**	X	X	**X**	X	X	X	**X**	X	X	X	X	X				

References

1. Brown JH, Jardetzky T, Saper MA, Samraoui B, Bjorkman PJ, Wiley DC. A hypothetical model of the foreign antigen binding site of class II histocompatibility molecules. Nature. 1988;332:845–50.
