# Supplementary material for: Alternated selection mechanisms maintain adaptive diversity in different demographic scenarios of a large carnivore
Source: BMC Evol Biol. 2019 Apr 11;19:90. doi: 10.1186/s12862-019-1420-5 (PMC6460805; doi:10.1186/s12862-019-1420-5)
Supplement: Supplementary file 6 — Table S6. Model selection information, including Akaike Information Criterion (AICc), delta AICc (ΔAICc) and weight of selected model for number of alleles (Na), allelic richness (AR), observed (Ho) and expected (He) heterozygosities. (PDF 128 kb) [file 12862_2019_1420_MOESM6_ESM.pdf]

Additional file 6

Syntax for linear mixed effect models was as follows:

m1: lmer (formula: diversity measure ~ 1 + (1|locus));

m2: lmer (formula: diversity measure ~ demographic group + (1|locus));

m3: lmer (formula: diversity measure ~ locus type + (1|locus));

m4: lmer (formula: diversity measure ~ demographic group + locus type + (1|locus));

m5: lmer (formula: diversity measure ~ demographic group + locus type + demographic group*locus type + (1|locus));

m6: lm (formula: diversity measure ~ 1);

m7: lm (formula: diversity measure ~ demographic group);

m8: lm (formula: diversity measure ~ locus type);

m9: lm (formula: diversity measure ~ demographic group + locus type);

m10: lm (formula: diversity measure ~ demographic group + locus type + demographic group*locus type).

Table S6. Model selection information, including Akaike Information Criterion (AICc), delta AICc (ΔAICc) and weight of selected model for number of alleles (Na), allelic richness (AR), observed (Ho) and expected (He) heterozygosities.

Diversity measure	Selected model	AICc	ΔAICc	Weight
Na	m2	442	0	0.433
	m4	442	0.46	0.345
	m5	443	1.35	0.220
AR	m4	297	0	0.803
He	m2	-102	0	0.656
Ho	m1	-53	0	0.405
	m8	-52	0.70	0.285
	m3	-51	1.21	0.221
