# Supplementary material for: Alternated selection mechanisms maintain adaptive diversity in different demographic scenarios of a large carnivore
Source: BMC Evol Biol. 2019 Apr 11;19:90. doi: 10.1186/s12862-019-1420-5 (PMC6460805; doi:10.1186/s12862-019-1420-5)
Supplement: Supplementary file 7 — Table S7. Estimates of combined diversity measures of each demographic group, standard errors (S.E.) and probability inferred from t-value, according to the selected model. (PDF 11 kb) [file 12862_2019_1420_MOESM7_ESM.pdf]

Additional file 7

Table S7. Estimates of combined diversity measures of each demographic group, standard errors (S.E.) and probability inferred from t-value, according to the selected linear mixed effects model.

Diversity measure	Selected model	Demographic group	Estimate	S.E	Probability
Na	m2 / m4 / m5	Persistent	5.476	0.225	**< 0.001**
		Expanding	4.315	0.248	**< 0.001**
		Isolated	3.310	0.246	< **0.001**
AR	m4	Persistent	4.853	0.424	**< 0.001**
		Expanding	4.606	0.126	0.05
		Isolated	4.223	0.125	**0.004**
He	m2	Persistent	0.646	0.025	< **0.001**
		Expanding	0.604	0.026	0.101
		Isolated	0.529	0.025	< **0.001**
