# Supplementary material for: Alternated selection mechanisms maintain adaptive diversity in different demographic scenarios of a large carnivore
Source: BMC Evol Biol. 2019 Apr 11;19:90. doi: 10.1186/s12862-019-1420-5 (PMC6460805; doi:10.1186/s12862-019-1420-5)
Supplement: Supplementary file 8 — Table S8. Mean number of alleles (Na), mean number of effective alleles (Ne), mean allelic richness (AR), observed (Ho) and expected (He) heterozygosities, and mean inbreeding coefficient (FIS) at the microsatellite and MHC loci, in three demographic groups (persistent, expanding and isolated), including standard deviation (s.d). The probability of the values to differ between microsatellite and MHC loci was tested by a one-tailed t-test (p-value). Values with statistical significance are in bold (p < 0.05). (PDF 28 kb) [file 12862_2019_1420_MOESM8_ESM.pdf]

Additional file 8

Table S8 Mean number of alleles (Na), mean number of effective alleles (Ne), mean allelic richness (AR), observed (Ho) and expected (He) heterozygosities, and inbreeding coefficient (F_{IS}) at the microsatellite and MHC loci, in three demographic groups (persistent, expanding and isolated), including standard deviation (s.d). The probability of the values to differ between microsatellite and MHC loci was tested by a one-tailed t-test (p-value). Values with statistical significance are in bold (p<0.05).

**Group**	**Microsatellites**	**MHC**	**p-value**
**Persistent**	**Mean (s.d.)**	**Mean (s.d.)**	
Na	5.5 (0.3)	5.7 (0.9)	0.421
Ne	3.2 (0.2)	4.1 (0.7)	0.153
AR	3.5 (0.9)	4.9 (1.0)	0.099
Ho	0.578 (0.027)	0.675 (0.017)	**0.003**
He	0.639 (0.028)	0.742 (0.048)	0.073
F_{IS}	0.098 (0.015)	0.085 (0.039)	0.391
**Expanding**			
Na	4.2 (0.2)	5.0 (0.6)	0.150
Ne	2.8 (0.1)	3.7 (0.5)	0.085
AR	3.2 (0.9)	4.8 (0.8)	**0.047**
Ho	0.561 (0.033)	0.813 (0.035)	**<0.001**
He	0.584 (0.030)	0.724 (0.034)	**0.010**
F_{IS}	0.040 (0.028)	-0.124 (0.008)	**<0.001**
**Isolated**			
Na	3.3 (0.2)	4.0 (0.6)	0.163
Ne	2.3 (0.1)	2.8 (0.5)	0.225
AR	2.9 (0.8)	4.0 (0.8)	0.098
Ho	0.546 (0.036)	0.733 (0.167)	0.189
He	0.523 (0.024)	0.613 (0.084)	0.199
F_{IS}	-0.049 (0.047)	-0.160 (0.131)	0.247
