# Supplementary material for: Alternated selection mechanisms maintain adaptive diversity in different demographic scenarios of a large carnivore
Source: BMC Evol Biol. 2019 Apr 11;19:90. doi: 10.1186/s12862-019-1420-5 (PMC6460805; doi:10.1186/s12862-019-1420-5)
Supplement: Supplementary file 9 — Table S9. Thermocycling profile for PCR amplification of the three MHC fragments used in this work. Annealing temperature (AT) was set initially at 64 °C (DRB1), 54 °C (DQA1) and 76 °C (DQB1). (PDF 97 kb) [file 12862_2019_1420_MOESM9_ESM.pdf]

Additional file 9

Table S9 Thermocycling profile for PCR amplification of the three MHC fragments used in this work. Annealing temperature (AT) was set initially at 64°C (DRB1), 54°C (DQA1) and 76°C (DQB1).

Temperature (°C)	Time	Number of cycles
95	10 min	
95	30 sec	12x
AT down 0.5°C per cycle	60 sec	
72	60 sec	
95	30 sec	24x
AT after touchdown	1 min	
72	1 min (DQA1 and DQB1)/30 sec	
72	10 min	
